# Perioperative evaluation of cardiac surgical risk: particularities in the emergency surgery – from the guidelines to the clinical practice

**Published:** 2013-09-25

**Authors:** AM Andronescu, AC Nechita, G Ittu, C Delcea, G Dumitrescu, MM Vintila

**Affiliations:** * 1st Internal Medicine and Cardiology Department, “Sfantul Pantelimon" Clinical Emergency Hospital Bucharest; ** "Carol Davila" University of Medicine and Pharmacy Bucharest

**Keywords:** perioperative evaluation, perioperative cardiac risk, pemergency surgery

## Abstract

** Rationale:** Cardiac risk in patients undergoing surgery depends on many factors from the patient's cardiovascular history to the surgical procedure itself, with its particularities, the type of anesthesia, fluid exchanges and the supervision of the patient. Therefore, this risk must be carefully considered and it determines the endorsement of perioperative measures with important medical implications.

** Objective:** Perioperative cardiac risk evaluation guidelines were published since 2010 and they represent a highly important assessmnet tool. Emergency surgery requires an adaptation of the guidelines to the actual medical situations in extreme conditions.

** Methods, Results, Discussion: **Analyzing the way the perioperative evaluation itself is conducted is an extremely important tool. Quantifying the clinical application of the guidelines, one can monitor real parameters and find solutions for improving medical care. The current study was conducted on a representative sample of 8326 patients, respecting the recommendation strategies for calculating the surgical risk adapted for the emergency surgery setting. The dominant conclusion is the need to develop a standardized form, summarized for quick and objective assessment of perioperative cardiac risk score. Only a complex medical team could calculate this score while the decisional team leader for the surgical patient remains the surgeon.

## Introduction

Once the guidelines for perioperative evaluation of cardiac risk in noncardiac surgical interventions were published, it seemed obvious that the team managing this risk was granted access to an elaborate scientific instrument. 

 However, the “guidelines", an instrument with advisory value, must be supplemented by a certain protocol specific to each medical institution. 

 Cardiac risk is not dependable only on “cardiac factors" but also on surgical ones, represented by the extension, duration and type of procedure that determines the changes in body temperature, blood losses and fluid exchanges [**1,2**]. 

The surgery per se determines a stress response mediated by neuroendocrine factors, response that actually modifies the oxygen consumption in the myocardial fibers. 

 At the same time, prothrombotic and fybrinolytic factors are modified, triggering a state of hypercoagulability directly proportional to the type and duration of the surgical intervention, especially in patients with associated pathology. 

 We must also mention the cardiac risk determined by the “anesthetic factors", depending on the duration of anesthesia, the anesthetic medication administered perioperatorive that, in turn, alters blood pressure, heart rate, myocardial oxygen consumption, especially considering the fact that metabolizing the anesthetic medication continues after the surgical procedure itself. 

 The permanent collaboration between the surgeon, anesthesiologist and cardiologist represents a sine qva non condition for an optimal perioperative outcome.

## Objectives

The aim of this paper is to identify the particularities of evaluating the perioperative cardiac risk in patients with non-cardiac surgical interventions specific to an emergency hospital, as well as to find solutions in the circumstances that the guidelines may not cover, when this is possible. 

## Methods

 We analyzed 8326 patients admitted consecutively to our hospital for surgical interventions from January 2010 to December 2012. We analyzed: 

 1. The assessment of emergent necessity of the surgical procedure 

 2. The assessment of the patient's hemodynamic instability 

 3. The assessment of the risk involved with the surgical procedure – assessed in accordance to the guideline recommendations [3,4] (**Table 1**) 

 4. Functional capacity measured in metabolic equivalents (METs) [5,6] 

 One MET represents the basal metabolic rate. 

 Without standardized testing, according to the guideline recommendations, the assessment was made by evaluating the capacity to undertake daily activities. 

 1 MET = energy consumption necessary in basal conditions 

 4 METs = energy consumption necessary to climb two flights of stairs 

 10 METs = allows the possibility to engage in sporting activity with high energy consumption (e.g swimming) 

5. The assessment of the cardiovascular risk itself according to the Lee Index [**7**] (modified Goldman) (**Table 2**) 

 6. ECG monitoring in preoperative as well as post-operative evaluation (evidence level IIa, IIb) [**8,9**] 

7. Establishing the perioperative risk scale according to the risk levels according to the guideline recommendations regarding each independent clinical factor equivalent to one point [**3,4,7**] (**Table 3**) 

**Table 1:** Cardiovascular risk corresponding to the type of intervention

Risk	Intervention
Low 1%	Breast, gynecological, urological minor, reconstructive minor
Medium 1-5%	Abdominal, head and neck, urological
High >5%	Intervention involves major intraabdominal vessels or peripheral vessels

**Table 2:** Lee index changes in determining perioperative risk

Clinical history	Low Risk	Compensated Medium Risk	Decompensated High Risk
Stable angina	-	x	x
Acute coronary syndrome	-	-	x
Heart failure	-	-	x
Stroke	-	x	x
Insulin-dependent diabetes	-	x	x
Renal disease	-	x	x
Age>	x	x	x

**Table 3:** Equivalence scale of the perioperative risk

Clinical history	Low Risk	Compensated Medium Risk
0	0.4	Minimum
1	0.9	Minimum
2	7	Medium
≥3	>11	High

 Evidence level I A.

 We then assessed the concordance between the level of perioperative cardiovascular risk and necessity of laboratory work was assessed. 

 8. The necessity of determining “risk biomarkers" [**10-13**]:

 - troponin

 - CRP

 - BNP

 - creatinine

 Recommendation with an evidence level - II A.

 9. Evaluation of left ventricular function – applicable only to those patients with very high risk (evidence level II A) [14,15]

 10. Applicability of a pharmacological strategy of perioperative risk reduction of one of the following classes of medication:

 A.Beta-blockers – scientific support according to the studies cited in the guidelines [**16-21**].

 B.Statins – recommended in the preventive therapy of cardiovascular pathology regardless of the degree of prevention [22,23].

 The analysis took into account the implicated risk of myopathy and rhabdomyolysis, symptoms that may be concealed by the administration of anesthetic and antialgic medication.

 C.Nitrates – no evidence was discovered that their use may reduce risk, even more, it is considered that their effect may be negative by reducing the preload, causing tachycardia and arterial hypotension [**24-25**].

 D.Angiotensin-converting-enzyme inhibitor (ACE inhibitor) – with positive effect especially for hypertensive patients [**26-27**]. Usually, the ACE inhibitor must be withheld 24 hours before surgery and restarted 24 hours after the intervention. 

 E.Diuretics – very frequently used, even if not explicitly in preventing cardiovascular complications, with an evidence level I [**28-31**].

 F.Aspirin – usually treatment with aspirin must be withheld if the risk of bleeding surpasses the cardiac benefit [32,33].

 G.Anticoagulants – their use must be appreciated according to the increased risk of bleeding, during and immediately after the surgical procedure [**34-36**]. 

 The analysis took into account the guideline recommendations for the anticoagulation protocol. The concordance with these recommendations is assessed in separate paper.

 For each of the ten established objectives we computed a percent of real applicability, which was then transformed into a grade on a scale from 0 to 10. Pharmacological strategy took into account the indications and contraindications of each drug, leading to a reduction of the grade corresponding to an administration beyond the recommendations.

 For each point mentioned above, the causes leading to the final score were analyzed.

## Results

 1. The assessment of emergent necessity of the surgical procedure

 The analysis comprised of all 8326 cases, each being individually assessed and the recommendation being established from the time of admittance to the hospital. The rightfulness in appreciating the surgical emergency was strictly correlated to the concordance between the preoperative and postoperative diagnosis. Discordance of diagnosis modified the initial appreciation of surgical emergency in 18.72% cases.

 This point was of course, appreciated exclusively by the surgeon.

 Score: 8.25.

 2. The assessment of the patient's hemodynamic instability

 The emergency physician, the intensive care physician and the cardiologist conducted this assessment. It was appreciated and recorded for all 8326 patients.

 Patient stabilization was initiated in the emergency room and continued in the intensive care department for 9.28% patients. Vital risk of the surgical decision established the decision in favor of the intervention in 33 (0.39%) unstable patients.

 Score: 10.

 3. The assessment of the risk involved with the surgical procedure 

 This evaluation was easily made in conformity with the guideline recommendations, as it is clearly deduced from the type of surgical procedure. 

 Score: 10.

 4. Functional capacity measured in metabolic equivalents

 This assessment was carried out only for the patients for whom a cardiovascular examination was performed, the group generally consisting of patients with medium and high surgical risk. 

 The Cardiology consult was performed in 4902 (58.87%) patients. Actual records of the calculated score were found for 1807 (21.7%) patients, since no validated protocol with specific directions existed.

 Score: 4.0

**Graph 1:**
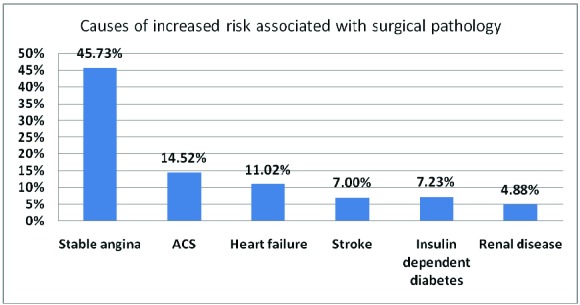
Causes of increased risk associated with surgical pathology

 5. The assessment of perioperative cardiovascular risk was accomplished for 58.70% of all patients. Causes for increased risk are listed below (Graph 1). 

 6. ECG monitoring was achieved preoperatively in all patients. 

 Regarding the postoperative period, ECG tracings were obtained in only 47.5% of the patients presenting with symptoms susceptible to be caused by cardiac pathology. ECG tracings were not obtained for high-risk patients in the absence of suggestive symptoms.

 Given this setting, it is possible that part of the patients were experiencing cardiac symptoms that were interpreted to be induced by other causes, or that certain asymptomatic cardiac complications may have gone undiagnosed. This gives the ECG recordings much more importance in the postoperative period for patients with symptoms susceptible of being cardiac in nature as well as for patients with a high cardiac risk.

 Score: 4.75

 7. Establishing the perioperative risk scale according to the risk levels

 This scale was not recorded in any way in any of the cases. The appreciation of this scale was formulated indirectly, without it being documented in the patients' records, although it seemed necessary and, with high probability, this risk was referred to when the patients or their families were explained the surgical risk. These explanations constituted the base for signing the informed consent regarding the surgical procedure, signature found in the all the patients' records.

 Score: 5.00

 8. The necessity of determining "risk biomarkers" 

 These biomarkers were determined for a number of 1207 (14.49%) patients, except for creatinine, which was determined, practically in 100% of cases.

 However, the analysis of biomarker measurements should be reported for patients with cardiac risk, depending on the associated pathology or perioperative evolution, determining a percentage change in 79.9% patients.

 Score 7.99

 9. Evaluation of left ventricular function

 This evaluation was determined according to the risk level for 1705 patients. Correlating the measurements with the actual need of assessment showed that this determination was made for 44.77% patients.

 Score: 4.47

 10. Applicability of a pharmacological strategy of perioperative risk reduction

 Requires a more complex discussion for each medication separately. Data are included in a separate analysis.

**Graph 2:**
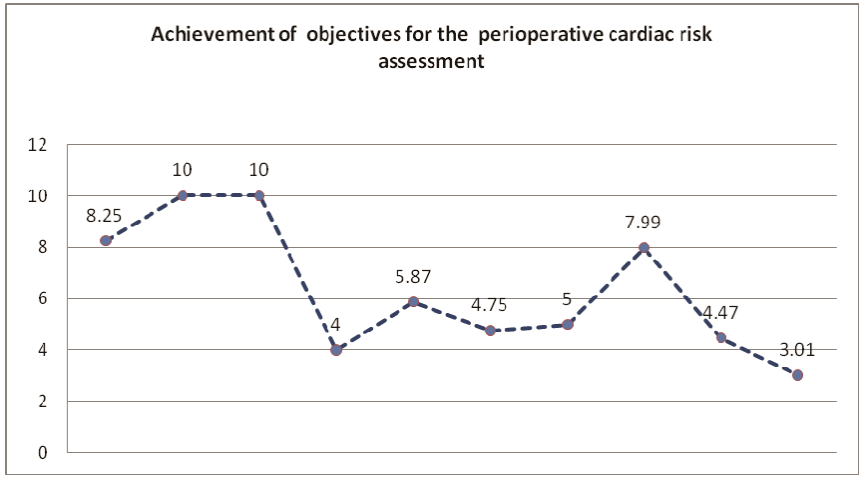
Achievement of objectives for the perioperative cardiac risk assessment

 Compared to the necessity of treatment, the pharmacological perioperative therapy was used as it follows:

 • beta-blocker: 59.81%

 • diuretic: 78.7%%

 • angiotensin-converting-enzyme inhibitor: 53.22%

 • inadequate use of nitroglycerin: 29.27% (its use outside the indication represents a negative score)

 • anticoagulant: 59.89%

 • antiplatelet 73.34%

 • statin: 5.24% (Graph 2)

 Score: 3.01

**Graph 3:**
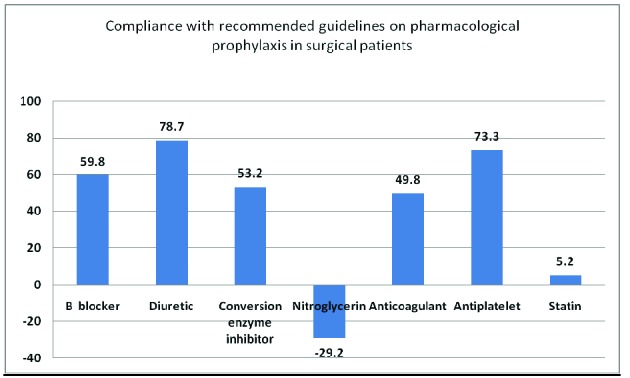
Compliance with recommended guidelines on pharmacological prophylaxis in surgical patients

## Conclusions

 Perioperative cardiac risk assessment is an obvious need to appreciate the appropriateness and type of surgery.

 Knowledge of the quantified cardiac risk significantly influences medical thinking. Although the guidelines on "Pre-operative Cardiac Risk Assessment and Perioperative Cardiac Management in Non-Cardiac surgery" have been developed by the European Society of Cardiology and adopted by the Romanian Society of Cardiology since 2010, they were not succeeded by the development of a standardized protocol.

 It is necessary to develop a standardized, synthesized form, no longer than one page, including predetermined answers to all important points of the guidelines. This way we could appreciate objectively, quickly and easily the score for perioperative cardiac risk.

 Patients with cardiac risk require a careful post-operative surveillance even in the absence of cardiac symptoms to influence the pharmacological decision, intra-hospital evolution and length of stay, this type of assessment representing a way to detect weaknesses and necessary adjustments for the favorable outcome desired by everyone. Medical responsibility is a complex engagement and the decisional team leader for surgical patients is, ultimately, the surgeon.
